# Complete mitogenome of *Phthorimaea operculella* (Lepidoptera: Gelechioidea: Gelechiidae)

**DOI:** 10.1080/23802359.2021.1920861

**Published:** 2021-06-22

**Authors:** Yan-Fei Song, Chun-Guang Xiao, Shuai Ye, Bin Zhang, Mao-Fa Yang, Jian-Feng Liu

**Affiliations:** a Institute of Entomology, Guizhou University, Guiyang, China; Institute of Entomology, Guizhou University, Guiyang, China; Scientific Observing and Experimental Station of Crop Pest in Guiyang, Ministry of Agriculture, Guiyang, China; b Institute of Potato, Anshun Academy of Agricultural Sciences, Puding, China; c Plant Protection and Quarantine Station of Guiyang City, Guiyang, China; d College of Tobacco Science, Guizhou University, Guiyang, China

**Keywords:** Mitogenome, Gelechiidae, *Phthorimaea operculella*, phylogeny

## Abstract

In this research, the complete mitochondrial genome (mitogenome) of *Phthorimaea operculella* was sequenced and annotated. The mitogenome of *P. operculella* is 15,269 bp in length and contains 13 protein-coding genes (PCGs), 22 transfer RNA (tRNA) genes, 2 ribosome RNA (12s and 16srRNA) genes and 1 control region. In addition, we used *Endoclita signifier* as the outgroup to analyze phylogenetic relationship, and the phylogenetic tree showed the sister relationship between *P. operculella* and *Tuta absoluta.*

Potato tuberworm*, Phthorimaea operculella* (Lepidoptera: Gelechioidea: Gelechiidae), is a major pest of Solanaceae crops (Rondon 2010). This pest spreads all over the world especially subtropic, tropical, and Mediterranean regions, including China. It is difficult to control this pest because the female adults usually lay eggs in hiding places, such as plant tuber and foliage, meanwhile, the larvae can survive in both field and storage condition (Sporleder et al. 2004; Yuan et al. 2018). The larvae of *P. operculella* mainly feed on Solanaceae crops like potato, tobacco, and tomato. In China, the loss of average potato yield can reach 40–45% (Rondon and Gao 2018), which makes a considerable damage to the industry of potato. Previous studies mainly focused on the ecology, biology, and management of *P. operculella*, yet few people studied its molecular characteristic. Consequently, we determined to sequence the mitogenome of *P. operculella* to provide the fundamental information about its genome.

The larvae were collected from Puding county (105˚27′49″E, 26˚26′36″N), Guizhou, China, in November 2020 and reared in the laboratory of the Institute of Entomology, Guizhou University, Guiyang, China. The voucher specimen’s genome DNA and genitalia are deposited in the Institute of Entomology, Guizhou University, and the sample number is GUGC-Pht-00203 (Jian-Feng Liu, jianfengliu25@126.com). We used Qiagen DNeasy Blood and Tissue Kit (Cat. no. 69504) to extract the total DNA from a second instar larvae of *P. operculella*. The Illumina ReSeq library was prepared with an average insert size of 400 bp with 150 bp paired-end and then we used the Illumina Novaseq6000 platform (Berry Genomics, Beijing, China) to sequence it. To assemble and annotate complete mitogenome sequence, we used NOVOPlasty v2.7.2 (Dierckxsens et al. 2017) with K-mer value and MitoZ v2.3 (Meng et al. 2019) with default set, respectively. Finally, the annotation was adjusted by MITOS2 (Bernt et al. 2013) and Geneious Prime v2020.2.4 (Kearse et al. 2012).

The mitogenome of *P. operculella* (accession no. MW540822) is 15,269 bp in length, containing 13 PCGs, 22 tRNA genes, 2 rRNA genes, and 1 control region. The content of A + T is distinctly higher than that of G + C (A:39.5%; G:7.9%; C:11.5%; T:41.0%), which is similar to other Gelechiidae mitogenome (Yuan et al. 2019). The 16sRNA gene and 12sRNA gene is 1341bp and 808 bp long, respectively. Most of protein-coding genes initiated with ATN, and ended with TAA, except for *COX1* used CGA as the start codon and *COX1, COX2, ND5* used incomplete T as the stop codon. The length of 22 tRNA ranged from 64 bp (tRNA-T) to 71 bp (tRNA-K).

To figure out the evolutionary status of *P. operculella*, we downloaded 15 complete mitogenomes from GenBank database. Among which, *Endoclita signifier* was selected as the outgroup. The nucleotide sequence of 13 PCGs (remove the third codon position) and 2 rRNA genes were aligned using MAFFT v7.394 (Katoh and Standley 2013) with L-INS-I algorithm, and then poorly aligned results were removed by trimAl v1.4.1 (Capella-Gutiérrez et al. 2009). Under the best fitting GTR + I + G model, the phylogenetic tree was created from the maximum-likelihood method using the IQTREE v1.6.3 software (Nguyen et al. 2015). The result showed that *P. operculella* clustered with other seven Gelechiidae species, and it was part of the Gelechiidae (Figure 1).

**Figure 1.**
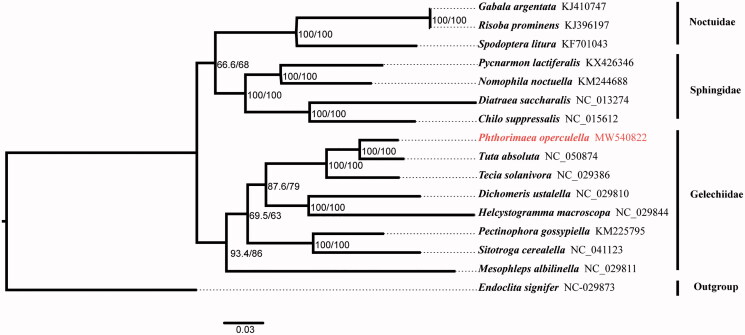
Maximum-likelihood phylogeny of 16 species based on concatenated nucleotide sequences of 13 PCGs (delete the third codon position). Number at nodes represent SH-aLRT support (%)/ultrafast bootstrap support (%) (1000 replicates).

## Data Availability

The data that support the findings of this study are openly available in NCBI at https:www.ncbi.nlm.nih.gov/, reference numbers KJ410747, KJ396197, KF701043, KX426346, KM244688, NC_013274, NC_015612, MW540822, NC_050874, NC_029386, NC_029810, NC-029844, KM225795, NC_041123, NC_029811, and NC_029873. The associated BioProject, BioSample, and SRA numbers are PRJNA699969, SAMN17817425, and SRR13741677, respectively (https://www.ncbi.nlm.nih.gov/sra/?term=SRR13741677).

## References

[CIT0001] Bernt M, Donath A, Jühling F, Externbrink F, Florentz C, Fritzsch G, Pütz J, Middendorf M, Stadler PF. 2013. MITOS: improved de novo metazoan mitochondrial genome annotation. Mol Phylogenet E. 69(2):313–319. 10.1016/j.ympev.2012.08.023 22982435

[CIT0002] Capella-Gutiérrez S, Silla-Martínez JM, Gabaldón T. 2009. trimAl: a tool for automated alignment trimming in large-scale phylogenetic analyses. Bioinformatics. 25(15):1972–1973. 19505945 10.1093/bioinformatics/btp348 PMC2712344

[CIT0003] Dierckxsens N, Mardulyn P, Smits G. 2017. NOVOPlasty: de novo assembly of organelle genomes from whole genome data. Nucleic Acids Res. 45(4):e18. 28204566 10.1093/nar/gkw955 PMC5389512

[CIT0004] Katoh K, Standley DM. 2013. MAFFT multiple sequence alignment software version 7: improvements in performance and usability. Mol Biol E. 30(4):772–780. 10.1093/molbev/mst010 PMC3603318 23329690

[CIT0005] Kearse M, Moir R, Wilson A, Stone-Havas S, Cheung M, Sturrock S, Buxton S, Cooper A, Markovitz S, Duran C, et al. 2012. Geneius basic: an integrated and extendable desktop software platform for the organization and analysis of sequence data. Bioinformatics. 28(12):1647–1649. 22543367 10.1093/bioinformatics/bts199 PMC3371832

[CIT0007] Meng GL, Li YY, Yang CT, Liu SL. 2019. Mitoz: a toolkit for animal mitochondrial genome assembly, annotation and visualization. Nucleic Acids Res. 47(11):e63. 30864657 10.1093/nar/gkz173 PMC6582343

[CIT0008] Nguyen LT, Schmidt HA, von Haeseler A, Minh BQ. 2015. IQ-TREE: a fast and effective stochastic algorithm for estimating maximum-likelihood phylogenies. Mol Biol E. 32(1):268–274. 10.1093/molbev/msu300 PMC4271533 25371430

[CIT0009] Rondon SI. 2010. The potato tuberworm: a literature review of its biology, ecology, and control. Am J Pot Res. 87(2):149–166.

[CIT0010] Rondon SI, Gao Y-L. 2018. The journey of the potato tuberworm around the world. In Perveen K, editor. Moths – pests of potato, maize and sugar beet. London (UK): IntechOpen.

[CIT0011] Sporleder M, Kroschel J, Gutierrez Quispe MR, Lagnaoui A. 2004. A temperature-based simulation model for the potato tuberworm, *Phthorimaea operculella* Zeller (Lepidoptera; Gelechiidae). Environ Entomol. 33(3):477–486.

[CIT0012] Yuan H-G, Wu S-Y, Lei Z-R, Rondon SI, Gao Y-L. 2018. Sub-lethal effects of *Beauveria bassiana* (Balsamo) on field populations of the potato tuberworm *Phthorimaea operculella* Zeller in China. J Integr Agr. 17(4):911–918.

[CIT0013] Yuan M, Yang H, Dai R-H. 2019. Complete mitochondrial genome of *Sitotroga cerealella* (Insecta: Lepidoptera: Gelechiidae). Mitochondrial DNA Part B. 4(1):235–236.

